# P_II_ Protein-Derived FRET Sensors for Quantification and Live-Cell Imaging of 2-Oxoglutarate

**DOI:** 10.1038/s41598-017-01440-w

**Published:** 2017-05-03

**Authors:** Jan Lüddecke, Liliana Francois, Philipp Spät, Björn Watzer, Tomasz Chilczuk, Gernot Poschet, Rüdiger Hell, Bernhard Radlwimmer, Karl Forchhammer

**Affiliations:** 1 Interfaculty Institute for Microbiology and Infection Medicine, Division Organismic Interactions, University of Tübingen, Tübingen, Germany; 2 Division of Molecular Genetics, German Cancer Research Center (DKFZ), Heidelberg, Germany; 3 Centre for Organismal Studies Heidelberg, Rupprecht-Karls-Universität Heidelberg, Heidelberg, Germany

## Abstract

The citric acid cycle intermediate 2-oxoglutarate (2-OG, a.k.a. alpha-ketoglutarate) links the carbon and nitrogen metabolic pathways and can provide information on the metabolic status of cells. In recent years, it has become exceedingly clear that 2-OG also acts as a master regulator of diverse biologic processes in all domains of life. Consequently, there is a great demand for time-resolved data on 2-OG fluctuations that can’t be adequately addressed using established methods like mass spectrometry-based metabolomics analysis. Therefore, we set out to develop a novel intramolecular 2-OG FRET sensor based on the signal transduction protein P_II_ from *Synechococcus elongatus* PCC 7942. We created two variants of the sensor, with a dynamic range for 2-OG from 0.1 µM to 0.1 mM or from 10 µM to 10 mM. As proof of concept, we applied the sensors to determine *in situ* glutamine:2-oxoglutarate aminotransferase (GOGAT) activity in *Synechococcus elongatus* PCC 7942 cells and measured 2-OG concentrations in cell extracts from *Escherichia coli in vitro*. Finally, we could show the sensors’ functionality in living human cell lines, demonstrating their potential in the context of mechanistic studies and drug screening.

## Introduction

The emerging field of metabolomics has the potential to accurately describe the physiological state of a cell at the time of measurement^1, 2^. Technological improvements during the last decade have led to more rapid and precise metabolite identification^3^; however, state of the art HPLC-MS based analyses require extraction of the metabolites from the cells, making the study of detailed temporal metabolite fluctuations a challenging task. Additionally, certain metabolites are not stable under commonly used extraction conditions or their recovery rates are low^4^. To overcome these drawbacks, a variety of genetically encoded, protein-based sensors were developed. These *in vivo/ex vivo* sensors allow real-time measurements of high temporal resolution without disrupting the cell. To date, there are hundreds of sensors available, not only for metabolites, but also for cellular aspects like ion concentration, mechanical stress, enzyme and kinase activity, redox potential, etc. (http://biosensor.dpb.carnegiescience.edu/)^5^. The majority of these sensors utilize the growing number of optimized fluorescence proteins (FPs) to create a Förster resonance energy transfer (FRET) readout^6, 7^. FRET occurs when donor and acceptor fluorophores with overlapping emission and excitation spectra come in close proximity. Following excitation of the donor, energy is transmitted to the acceptor in a non-radiative manner by means of intermolecular long-range dipole–dipole coupling and emitted by the acceptor. Ligand-binding-induced conformational changes in the sensors containing both, donor and acceptor fluorophres, results in altered FRET efficiency, which can be monitored under a fluorescence microscope or in a fluorimeter^8^.

One central metabolite of high interest is 2-oxoglutarate (2-OG), which links the carbon and nitrogen metabolic pathways in all domains of life. 2-OG is used as the carbon skeleton for nitrogen assimilatory reactions and has been proposed as a master regulatory metabolite^9^. It has been shown that the 2-OG pool reacts to changes in extracellular nitrogen availability within minutes and its half-life has been estimated as 0.5 s^10–13^. Apart from the regulatory P_II_ proteins (see below) 2-OG is sensed by a number of transcription factors^9^. Furthermore, 2-OG acts as a starvation signal in eukaryotes like *S. cerevisiae* or the metazoa *C. elegans* ^14, 15^. Competitive inhibition of 2-OG-dependent DNA and histone demethylases by the oncometabolite and 2-OG analogue 2-hydroxyglutarate have been shown to be the cause of cancer-specific epigenome and gene expression alterations in glioma and acute myelogenous leukemia^16–18^. Furthermore, 2-OG levels were proposed to regulate the epigenome of differentiating human pluripotent stem cells^19^ and embryonic stem cells^20, 21^. In mice it has recently been shown, that 2-OG acts as a systemic signal providing protection from cardiac ischemia^22^. In bacteria, the sugar-uptake phosphotransferase system (PTS) in *E. coli* is regulated by the 2-OG/phosphoenolpyruvate ratio^23, 24^. The PTS not only promotes sugar transport but is also responsible for activation or inhibition of the adenylate cyclase which produces cyclic AMP, a very important signaling molecule that affects the expression of a vast range of genes^25, 26^. These examples demonstrate the importance of 2-OG as a regulatory metabolite and underline the need for a functional sensor in living cells, which allows investigations of 2-OG fluctuations with high spatial and temporal resolution.

The small trimeric regulatory protein P_II,_ which is widely distributed in prokaryotes and chloroplasts, is known as a sensor of cellular 2-OG levels^27^. Binding of 2-OG leads to conformational changes in the protein structure in a concentration dependent manner^27–30^. These conformational changes modulate the interaction of P_II_ with its regulatory targets^31^. In previous studies, these interactions have been utilized to create inter-molecular FRET sensors employing cyanobacterial P_II_ proteins and their targets N-acetyl-L-glutamate kinase (NAGK) and PipX. These sensors have successfully been used to expand the knowledge about the 2-OG dependent mode of interaction between P_II_ and its targets^32–34^. However, FRET sensors using protein-protein interactions have disadvantages, especially for applications in living cells, where different expression rates and protein half-lives have to be taken into account, as well as the increased chance of unwanted side reactions, e.g. by NAGK enzymatic activity. Berg *et al*. have constructed a P_II_-based intramolecular FRET sensor to read out fluctuations in ATP/ADP levels, after having inactivated the 2-OG binding site in P_II_^35^. This motivated us to create an enhanced intra-molecular 2-OG FRET sensor making use of the high specificity of P_II_ proteins towards 2-OG.

As a first application we used the 2-OG sensor for an *in situ* glutamine:2-oxoglutarate aminotransferase (GOGAT) assay. GOGAT catalyzes the reductive transfer of the amide group from glutamine to the carbon backbone of 2-OG, which yields two molecules of glutamate. This is a key reaction in nitrogen assimilation in bacteria and plants^36^, but studies on GOGAT activity regulation are scarce^37^, due to the lack of a simple assay. Using the P_II_-based 2-OG specific FRET sensors, we present here the determination of the *in situ* fdGOGAT activity in the unicellular cyanobacteria *Synechococcus elongatus* PCC 7942 (hereafter designated as *S. elongatus*). Furthermore, we used the sensor to determine 2-OG concentrations in cell extracts from *E. coli*. Finally, we conducted the first application of a real-time 2-OG sensor in mammalian cells to measure 2-OG fluctuations in human glioblastoma brain-cancer cells and other cell lines. These experiments provide the proof of principle that P_II_-based 2-OG specific FRET sensors could become useful tools for studying cancer patho-mechanisms and as functional readouts in metabolic-drug screening.

## Results and Discussion

### Development of the FRET Sensor

On our attempt to construct a 2-OG sensor that employs the conformational change of P_II_ upon 2-OG binding, we made use of the knowledge gained during the development of the P_II_-NAGK FRET sensor^32^ and crystal structures of *S. elongatus* P_II_^28^. Different sensor variants were constructed, most of which use the monomeric (m) cyan FP mCerulean as the FRET donor and the yellow FP Venus as acceptor. The simplest approach was to fuse these FPs to the N and C-terminus of P_II_. Crystal structures of P_II_ with Mg^2+^-ATP + 2-OG bound display a conformational change in the C-terminus^28^: in the ligand free state (PDB: 1QY7) or while interacting with NAGK (PDB: 2V5H), the P_II_ C-terminus adopts a stretched conformation, pointing away from the trimer. By contrast, upon binding of Mg^2+^-ATP + 2-OG, the C-terminus retracts and folds over the metabolite binding site of the inter-subunit cleft (PDB: 2XUL). With the first variants, we aimed to asses if this conformational change in the C-terminus could be used to create a change in FRET.

**Figure 1:**
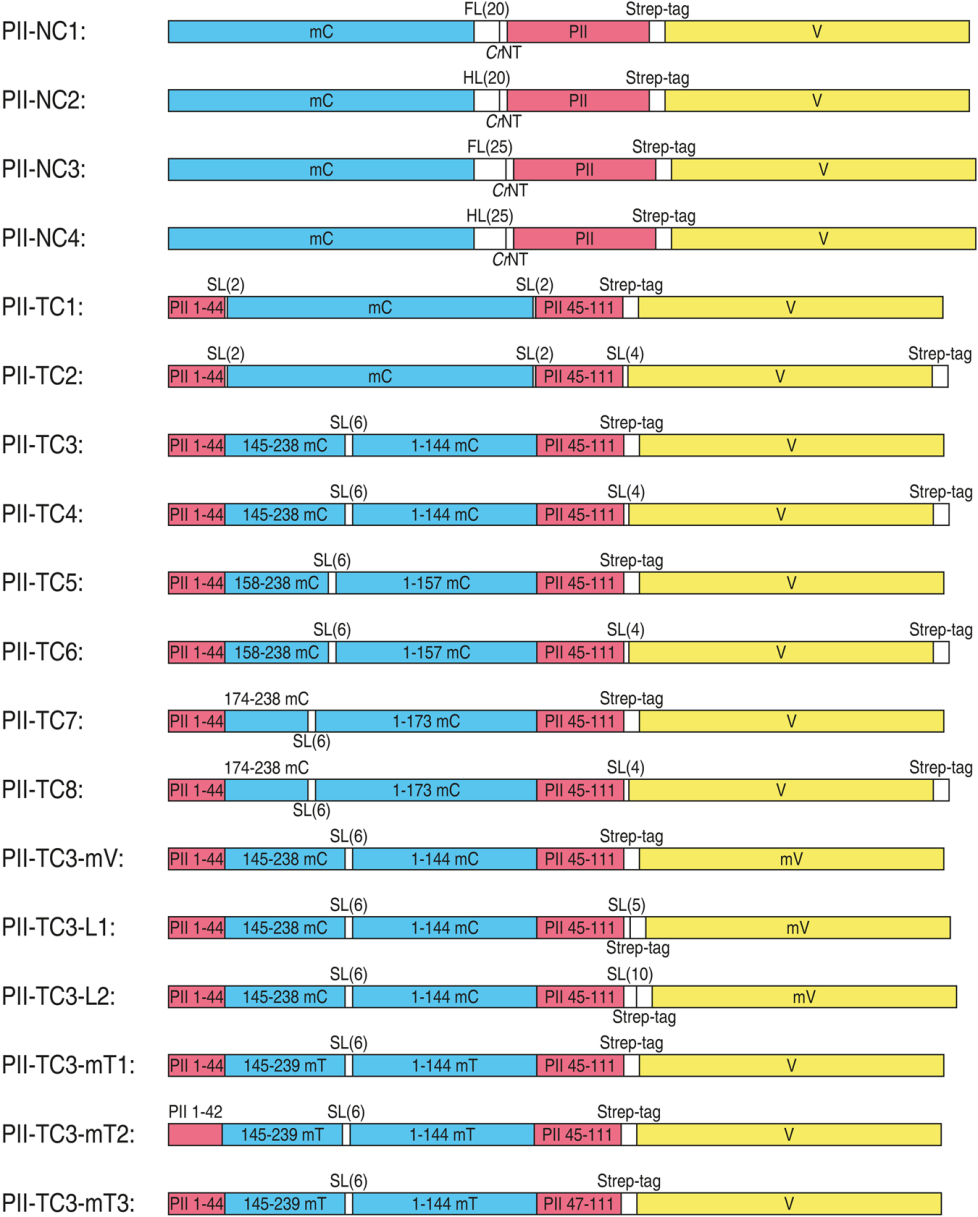
Schematic representation of the FRET-sensor constructs. Red: P_II_; Blue: mCerulean; Yellow: mVenus; White: linker regions. Flexible (FL) or stiff helical (HL) linkers or short linkers (SL) of different length, *Chlamydomonas reinhardtii* P_II_ N-terminus (*Cr*NT) or streptavidin affinity tag (Strep-tag). All Domains are presented to scale.

To achieve this goal, we had to overcome a problem associated with modifying the N-terminus of bacterial P_II_ proteins. The N-terminus of *S. elongatus* P_II_ is hidden within the protein and a direct fusion to the buried end leads to misfolding and degradation (data not shown). To solve this problem, we used the non-conserved extended N-terminus of *Chlamydomonas rheinhardtii* P_II_ that protrudes from the protein^38^ and fused these six N-terminal amino acids (S-A-F-P-G-V) to the N-terminus of *S. elongatus* P_II_. This indeed led to a stably expressed protein. On top of that, we added flexible (FL; L[SGGGG]_n_SAAA) or stiff helical (HL; A[EAAAK]_n_A) linkers of 20 or 25 amino acids (Fig. 1, P_II_-NC1 to 4)^39^. The Venus FP was fused to the C-terminus of P_II_ via the 12 amino acid long streptavidin affinity tag (Strep-tag), as has been done previously^32^. The Strep-tag should enable the transfer of the proposed C-terminal movement to the FP and was also used for protein purification. While P_II_-NC1 and NC3 could be purified in good quantity and quality, P_II_-NC2 and NC4 were not expressed in *E. coli*. Probably the helical linker led to misfolding and degradation of these proteins.

In other variants, we aimed to use the conformational change of the T-loop upon Mg^2+^-ATP + 2-OG binding to obtain a change in FRET. As shown in the crystal structures mentioned above, the stretched T-loops of ligand-free P_II_ are retracted and folded upon binding of Mg^2+^-ATP + 2-OG. This folding also prevents the interaction of P_II_ with other receptor proteins like NAGK or PipX^30^. We devised eight variants to test this approach. In the first two variants (Fig. 1, P_II_-TC1 and TC2) we inserted mCerulean with its natural C- and N-terminus into the tip of P_II_’s T-loop between amino acids 44 and 45. Venus was again placed at the C-terminus using either the Strep-tag, or a four amino acid linker (LAAA). FRET is not only influenced by the distance between donor and acceptor, but also by their angle towards each other^40^. With that in mind, we also used circularly permuted (cp) versions of mCerulean, by connecting its native C- and N-terminus with a short flexible linker (GSGGTG) and using other positions for the fusion into the T-loop, similar to the approach described by Berg *et al*. for the construction of a P_II_-based ATP/ADP sensor^35^. Topell *et al*. showed that circular permutations of GFP to positions Y145-N144, K158-Q157 as well as G174-D173 yielded correctly folded FPs^41^. We used these locations for fusion with the T-loop and again varied the linker between the P_II_ C-terminus and Venus (Fig. 1, P_II_-TC3 to 8).

All tested variants showed strong initial FRET signals, indicating that the FPs were correctly folded and in close proximity. Subsequently 0.1 mM ADP, 0.1 mM ATP or 0.1 mM ATP together with 1 mM 2-OG were added to the solution and the FRET change was measured (Table 1). Most variants showed only small FRET changes, either slightly increasing or decreasing the FRET upon effector molecule addition. Only P_II_-TC3 showed an 18% drop in FRET. The decreased FRET indicates that the FPs move away from each other, which is consistent with the crystal structures, where the T-loop folds downward upon 2-OG binding, away from the C-terminus.

**Table 1:** FRET change after addition of metabolites.

Sensor	% FRET change after addition of		
	ADP	ATP	ATP + 2-OG
P_II_-NC1	n. d.	0.3	4.4
P_II_-NC3	n. d.	3.1	3.1
P_II_-TC1	−2.0	−1.1	−1.7
P_II_-TC2	−1.1	1.0	5.7
P_II_-TC3	0.8	−0.8	−18.3
P_II_-TC4	−0.4	0.8	−2.5
P_II_-TC5	−6.1	−0.1	−3.1
P_II_-TC6	1.0	0.2	3.4
P_II_-TC7	−0.6	−0.8	−2.3
P_II_-TC8	6.2	0.8	0.9
P_II_-TC3-mV	n. d.	n. d.	−15.1
P_II_-TC3-L1	n. d.	n. d.	−15.4
P_II_-TC3-L2	n. d.	n. d.	−15.6
P_II_-TC3-mT1	n. d.	n. d.	−8.3
P_II_-TC3-mT2	n. d.	n. d.	25.8
P_II_-TC3-mT3	n. d.	n. d.	−13.3

FRET of the purified senor proteins was measured before and after addition of 0.1 mM ADP, 0.1 mM ATP or 0.1 mM ATP + 1 mM 2-OG and the relative FRET change was calculated. (n. d., not determined).

Since P_II_-TC3 showed the strongest FRET change so far, we tried further modifications of the P_II_-TC3 variant. GFPs have the tendency to dimerize. To allow the C-terminally attached Venus more flexibility in movement, we added the monomer mutation A206K^42^ and also elongated the linker between P_II_ and mVenus by five or ten amino acids (Fig. 1, P_II_-TC3-mV, -L1 and -L2). Even though P_II_-TC3-mV showed a higher initial FRET value, the relative FRET drop upon addition of ATP + 2-OG was lower compared to P_II_-TC3. Also the modified linkers did not increase the sensors signal (Table 1). Next, we tried to enhance the TC3 sensor by replacing mCerulean by the advanced mTurquoise2, which shows exceptional quantum yield, brightness, photostability and a fast maturation rate and is thus proposed as an optimal donor for FRET systems^43^. Additionally, we created two versions with partially deleted T-loops (P_II_-TC3-mT1 to 3). P_II_-TC3-mT1 and -mT3 showed lower signal changes than P_II_-TC3. Interestingly, P_II_-TC3-mT2, with T-loop amino acids 43 and 44 deleted, showed increased FRET upon addition of ATP + 2-OG. This indicates that the small deletion in the T-loop alters the orientation of the attached FP, demonstrating once again that subtle changes can have a dramatic and unpredictable impact on the signal output of a FRET sensor. A detailed analysis showed that P_II_-TC3-mT2 was only reacting to high 2-OG concentrations and was not as sensitively responding towards 2-OG as P_II_-TC3 (data not shown).

**Figure 2:**
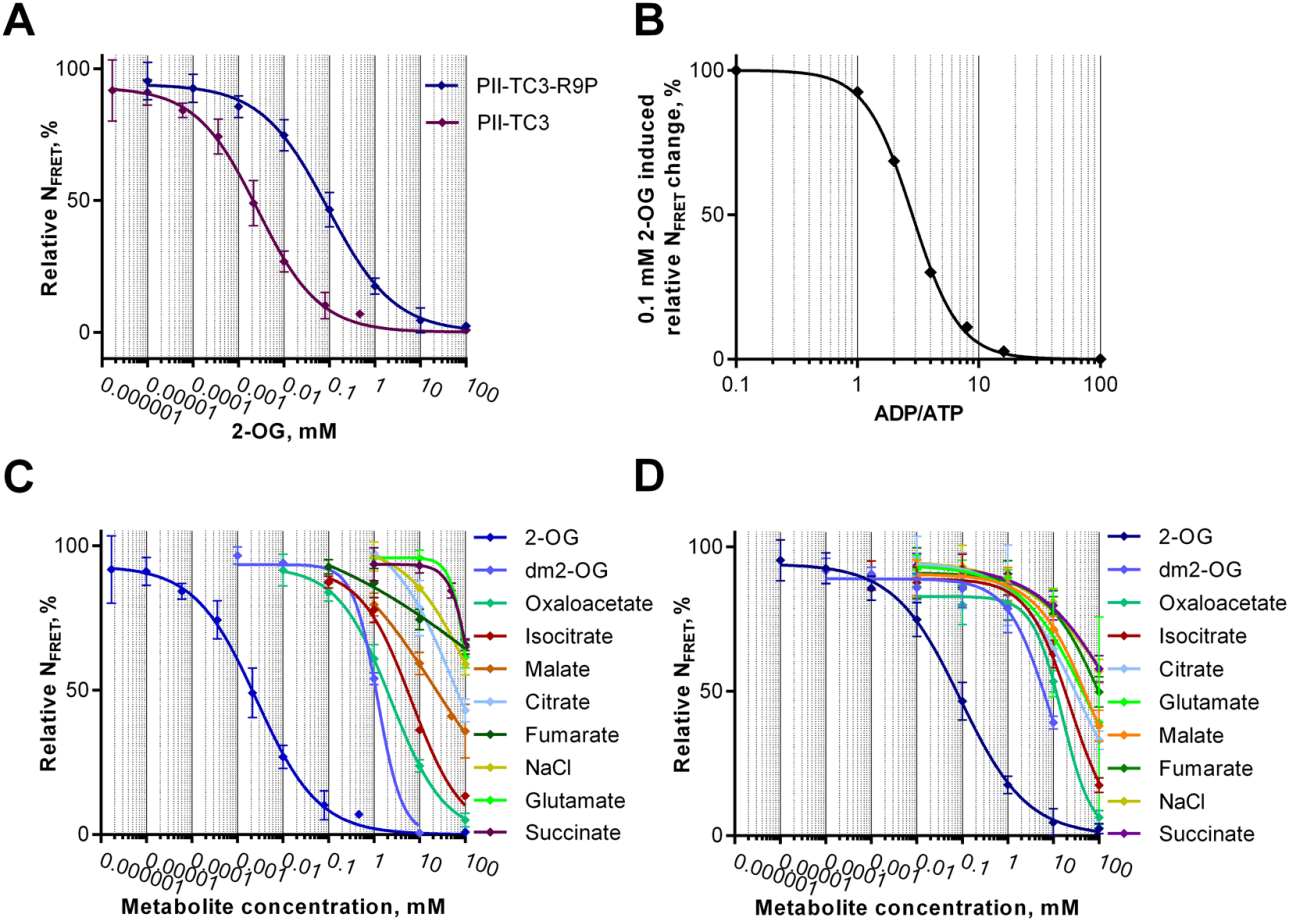
Properties of FRET-sensor P_II_-TC3. (**A**) Relative N_FRET_ response of P_II_-TC3 and P_II_-TC3-R9P to increasing 2-OG concentrations. (**B**) Influence of different ADP/ATP ratios on the N_FRET_ response of P_II_-TC3 upon addition of 0.1 mM 2-OG. (**C** and **D**) Relative N_FRET_ response of P_II_-TC3 (**C**) and P_II_-TC3-R9P (**D**), to different metabolites of the TCA cycle. Error bars: ±s.e.m., n = 4.

After all, P_II_-TC3 appeared to be the most promising sensor variant for sensitive 2-OG detection. To biochemically characterize P_II_-TC3, the affinity-tag isolated protein was further purified by size-exclusion chromatography to remove degradation products containing only one of the two FPs. We also optimized the buffer conditions and the workflow to improve TC3’s signal to noise ratio and to be able to use a 96-well plate setup and a small reaction volume. As previously described for P_II_-NAGK interactions, the proteins can be unstable when buffer conditions are quickly changed^32^. When the sensor, stored in a 50% glycerol buffer at −20 °C, was quickly mixed with the measurement buffer, it showed a lower signal to noise ratio and sensitivity, possibly due to dissociation of the trimer. To circumvent this problem, we tested a variety of reaction-buffer setups and came up with a mixture containing 15% glycerol, which not only stabilized the sensor and enabled easy preparation of a master mix for 96-well plate application, but also lead to a increase of the signal-to-noise ratio to 25%. Figure 2A shows the 2-OG induced FRET change under these optimized conditions. P_II_-TC3 displayed a K_d_ for 2-OG of approximately 3 µM. This is close to the binding constant of the high affinity 2-OG binding site in wild-type P_II_ protein, showing that, despite the insertion of mCerulean into the T-loop, P_II_-TC3 acts similar to P_II_-wildtype. However, for possible *in vivo* applications, where one- to two orders of magnitude higher 2-OG concentrations are expected, this response is too sensitive. Therefore, we constructed a variant of P_II_-TC3 with a mutation in the vicinity of the 2-OG binding site. Previously, it was shown that mutation of Arg9 reduces the affinity of 2-oxoglutarate binding^28^. Here, a mutation of arginine 9 to proline (R9P) decreased the affinity of P_II_ for 2-OG 35 times, resulting in a K_d_ of 91 µM (Fig. 2A). With a dynamic range of the FRET response from 10 µM to 10 mM, this sensor seems to be ideally suited to span the physiological relevant range of 2-OG concentrations. Because ATP and ADP compete for the same binding site on the P_II_ protein, but only ATP enables the binding of 2-OG, we also measured the competitive effect of ADP on the signal output. As shown in Fig. 2B, physiological ATP/ADP ratios (ATP > ADP) do not influence 2-OG sensing.

In the next step, we tested the sensitivity and specificity of TC3 and TC3-R9P. All metabolites of the tricarboxylic acid (TCA) cycle and the 2-OG analog dimethyl-2-OG (dm2-OG) were tested. As shown in Fig. 2C, TC3 is about 460 times more sensitive for 2-OG than for dm2-OG, which gave the second strongest signal. The third strongest signal was induced by oxaloacetate, with a 900 times reduced sensitivity. All other tested metabolites were even less interfering than oxaloacetate, demonstrating the high specificity of the sensor towards 2-OG. Interestingly, increasing NaCl concentrations also lead to a decrease in FRET. Most of the tested compounds were sodium salts or had to be titrated with NaOH to adjust the pH, and therefore might give a stronger response than pure compounds. Similarly, TC3-R9P was also highly specific for 2-OG. It showed an 85 times decreased sensitivity for dm2-OG and responded to other metabolites only at non-physiologically high concentrations (Fig. 2D).

### Establishing an *in situ* fdGOGAT assay using the 2-OG FRET-sensor

One possible application of a 2-OG FRET-sensor is the *in situ* determination of GOGAT activity. In contrast to previously described approaches, in which the formation of glutamate or consumption of glutamine were measured using HPLC-based amino acid quantification methods^44^, the FRET sensor based 2-OG quantification is much faster and cost efficient.

The *in situ* GOGAT assay was established for *S. elongatus*, which only possesses a ferredoxin-depending GOGAT. The method developed in the present study is based on experiments from Marqués *et al*. and Kameya *et al*.^44–46^. In short, the cells were permeabilized by toluene, the GOGAT substrates 2-OG, glutamine and reductant were added and the mixture incubated at 32 °C. The efficiency of cell permeabilization was tested via fluorescence microscopy using a live/dead staining kit for bacteria, revealing a very efficient permeabilization (Fig. S1). In our experimental setup, the natural reductant ferredoxin was substituted by methyl viologen as electron donor, which was reduced by addition of sodium dithionite. To avoid air-oxidization of the dithionite, we covered the reaction mix with paraffin oil. Samples were taken in 5 to 10 minutes intervals and the reaction was stopped by aeration through intense vortexing and cooling on ice, which is quick and easy to apply. Afterwards, the cells were separated by centrifugation and the supernatant was used for 2-OG quantification via FRET measurements. The components of the GOGAT-reaction mixture itself had no influence on the FRET measurement, since addition of the reaction mix to a 2-OG standard curve did not affect the read-out (Fig. S2).

**Figure 3:**
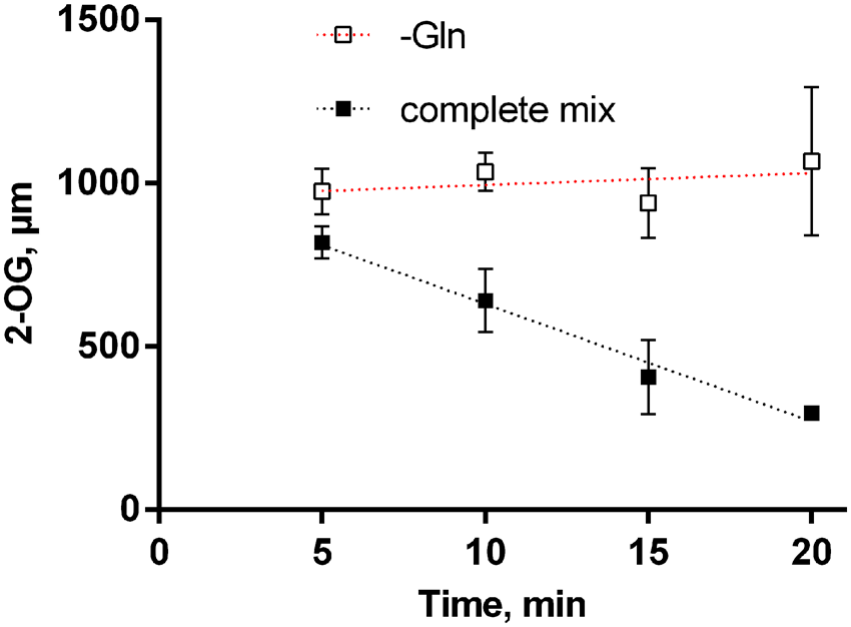
fdGOGAT assay with complete and incomplete reaction mix. *S. elongatus* cells were grown to an OD_750_ of 0.3 in nitrate-supplemented medium. The 2-OG concentrations in the reaction mixture was determined by FRET over time. Results are shown for a complete reaction mix (black squares and black dashed line) and the blank mix lacking glutamine (white squares and red dashed line). Error bars: ±s.e.m., n = 3.

When all components, including permeabilized cells, glutamine, 2-OG and the electron donor were present in the reaction mix, a decrease of the 2-OG concentration over time could be detected (Fig. 3). Preliminary experiments indicated a linear decline in the first 20 min of the reaction (data not shown) when using 4 * 10^8^ cells (≙6.7 mL of culture at OD_750_ = 0.3). When glutamine was omitted from the reaction mix, the concentration of 2-OG remained constant (Fig. 3), which indicates the absence of background 2-OG consuming side reactions.

### Characterization of fdGOGAT in *S. elongatus* using the *in situ* fdGOGAT Assay

**Figure 4:**
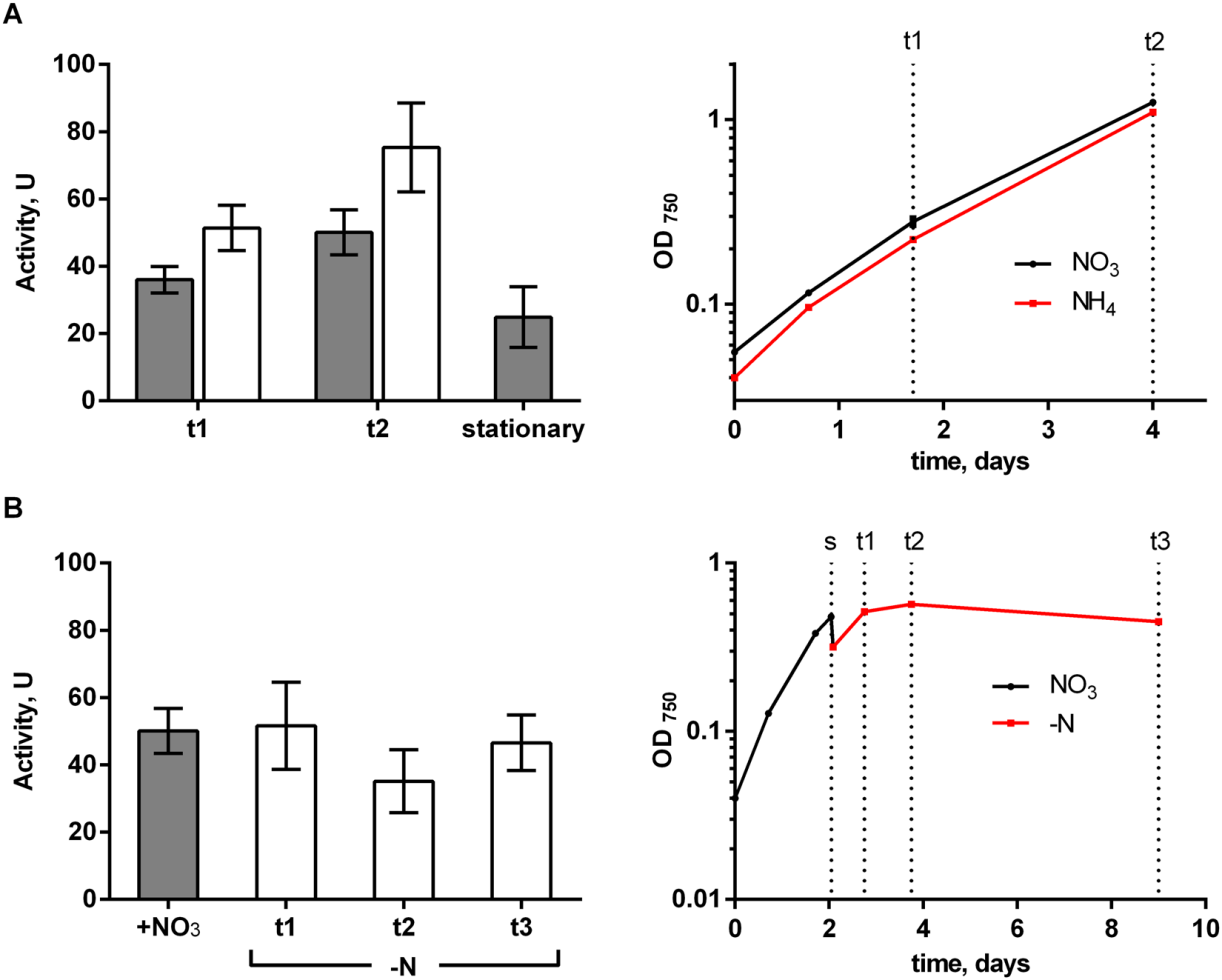
*In situ* fdGOGAT activity with different nitrogen sources. (**A**) *In situ* fdGOGAT activity of *S. elongatus* cultivated with nitrate (grey bars) or ammonium (white bars). Corresponding growth curves are shown on the right. Stationary cells were cultivated for 9 days to an OD_750_ of 5.5 to 6.0. (**B**) fdGOGAT activity of *S. elongatus* cultivated with nitrate and after shift (s) into nitrogen free medium (t1-3) with corresponding growth curve on the right. Error bars: ±s.e.m., n = 4.

The glutamine synthetase (GS) - GOGAT cycle represents the connection between the carbon and nitrogen metabolism in bacteria and plants. Whereas GS activity is easy to measure, established GOGAT activity assays are highly elaborate. To determine the fdGOGAT activity with the newly developed FRET-based assay *in situ*, we used, as a test case, *S. elongatus* cells grown in presence of nitrate or ammonia and cells that had been shifted to medium without nitrogen sources (Fig. 4). Samples were taken at three different phases of growth.

With nitrate as nitrogen source, the *in situ* fdGOGAT activity increased by approximately 40% from the early exponential (t1) to the late exponential growth phase (t2, Fig. 4A). After the cells entered the stationary phase, fdGOGAT activity decreased to approx. 70% of the initial value, probably in response to the reduced growth rate and declining metabolic activity in the stationary phase. Under ammonium supplemented growth conditions, the fdGOGAT activity was generally 40–50% higher as compared to nitrate conditions (Fig. 4A). Like in the nitrate-supplemented medium, a 47% increase in activity occurred from early exponential to late exponential growth phase.

The higher *in situ* fdGOGAT activity under ammonium-supplemented growth shows that fdGOGAT is regulated differently to GS. In cyanobacteria and many other prokaryotes, GS is subjected to tight regulation^36^. In *Synechocystis sp*. PCC 6803, GS activity is tuned down by addition of ammonia, both at the transcriptional level via the NtcA regulatory system^47^ as well as post-translationally by interaction with two inactivation factors IF7 and IF17^36^. Inhibition is reversed by ammonia depletion within 10–20 min. The ammonium-triggered short-term GS inactivation is essential for maintenance of glutamate homeostasis^48^. When ammonium is in abundance, although GS activity is decreased, more glutamate can be converted to glutamine. Under these conditions, a higher fdGOGAT activity could keep the glutamine/glutamate balance in equilibrium.

Under nitrogen starvation conditions (Fig. 4B), the fdGOGAT activity remains at a constant level. This level is kept constant even after 8 days of starvation, when the cells became chlorotic. This basal activity could ensure sufficiently high nitrogen assimilation capacity via the GS-GOGAT cycle, enabling the chlorotic cells to immediately assimilate any ammonia that becomes available in the environment.

### Determination of 2-OG Concentrations in Cell Extracts

Another possibility to use our sensor is to estimate the concentrations of 2-OG in cell-extracts. Metabolite concentrations in living cells are prone to fluctuations and can change very quickly during cell treatments, such as cell harvesting. To extract physiologically relevant quantities of metabolites form cells, the quenching of cellular activity before metabolite extraction is required^2^. There are several established metabolic inactivation methods. The use of −40 °C cooled 70% methanol rapidly freezes the cells, but also leads to metabolite loss^49^. Fast centrifugation and subsequent freezing works for heterotrophically growing cells, but is problematic for autotrophically growing cyanobacteria, which quickly adapt their metabolism to darkness. Fast filtration with illumination can solve this problem^50^. After quenching of enzymatic activities, the metabolites are immediately extracted from the cells. There are several established methods, but not all of them are suitable for 2-OG^4^: classical methods use mixtures of chloroform, methanol and buffer with 53% 2-OG recovery^51^, boiling ethanol with 58% recovery^52^ or pure −40 °C methanol with 82% recovery^4^. These protocols all include lyophilization of the extracts.

**Figure 5:**
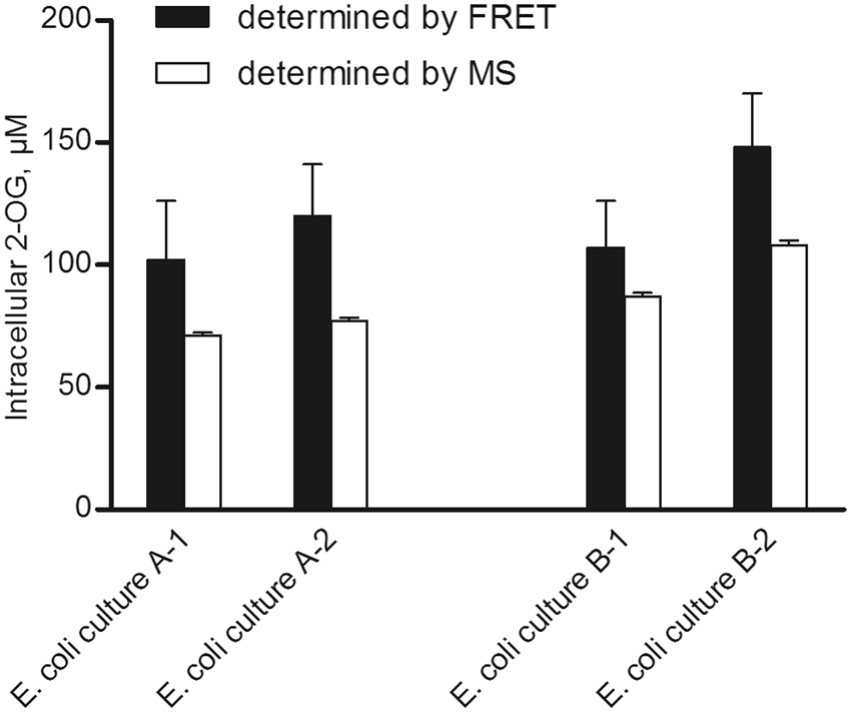
Intracellular 2-OG concentrations of *E. coli* 20 mL of exponentially growing *E. coli* culture were rapidly cooled to 0 °C and harvested by centrifugation. Two *E. coli* cultures (**A**,**B**) were used and two samples were taken per culture (1, 2). 2-OG was extracted and intracellular concentrations determined by FRET-sensor (grey; error bars: ±s.e.m. with n = 4) or GC-MS measurements (white; error bar denotes the standard error of the 2-OG calibration curve).

Our first approach was to rapidly filter the cells and immediately freeze the filters in liquid nitrogen. For this purpose, we used 2.5 cm diameter polyether sulfone (PES) membrane filters with a 0.45 µm pore size. The filters were immediately frozen in liquid nitrogen and afterwards briefly grounded. 2-OG was extracted with pure −40 °C methanol or with a mixture of methanol, chloroform and buffer. However, the organic solvents extracted unidentified compounds which interfered with the fluorescence measurements, showing high background fluorescence of more than 50% of the signal. Therefore, we used toluene to permeabilize the cells, after filtration and freezing as above, and measured 2-OG directly in the supernatant using P_II_-TC3. The cells were vortexed with 1400 rpm for 8 min at 25 °C in the presence of 1% toluene and 4% EtOH (v/v), similar to the protocol described above^46^. However, the standard deviations were still high. Therefore, we tried an even milder approach without freezing, by sampling the cultures into ice-cooled medium and thus immediately cooling the cells to 0 °C. The cells were then harvested by centrifugation and thereafter quickly permeabilized by toluene/EtOH, yielding reproducible results. The calculated intracellular 2-OG concentrations are shown in Fig. 5. In parallel to the FRET measurements, we determined the 2-OG concentrations by gas chromatography - mass spectrometry (GC-MS), revealing in general 20–30% lower values (for technical detail see Supplementary Information). This is likely due to losses during lyophilization and sample preparation for the GC-MS analyses. The measured intracellular 2-OG concentrations for *E. coli* cells are around 100 µM, using M9 medium with 19 mM NH_4_Cl (Fig. 5). These concentrations are in the same range as reported earlier for *E. coli* growing with high nitrogen supply^10, 11^. Despite this progress, quantification of cellular 2-OG levels from cell extracts remains problematic due to uncertainties in the extraction method^13^. The ultimate goal is therefore *in vivo* detection of cellular 2-OG levels in single cells. For this purpose, the TC3-R9L variant seemed promising and in the following, we applied this sensor to human cell lines, for which microscopic detection of metabolite FRET sensors is documented^53^.

### *Ex vivo* Measurements of 2-OG Fluctuations in Human Cell Lines

**Figure 6:**
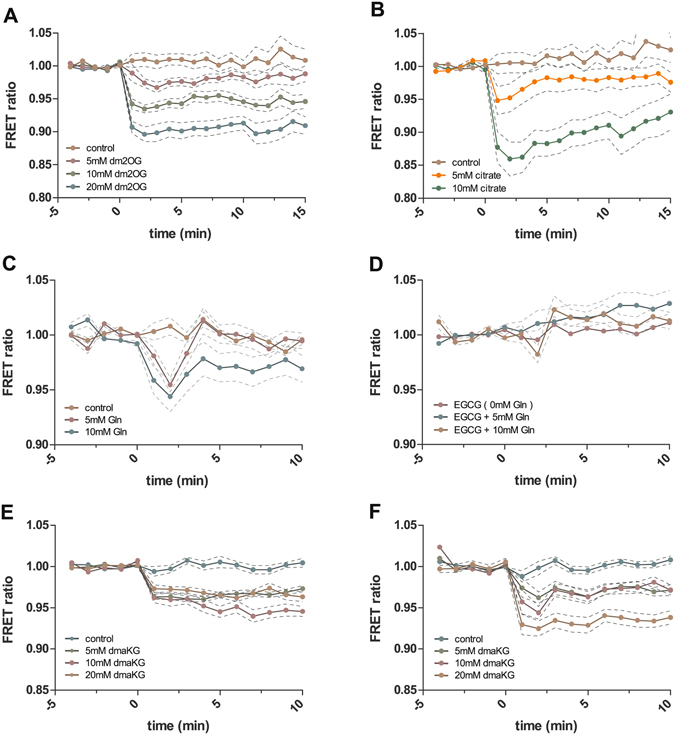
Effect of TCA-cycle metabolites on the FRET signal of TC3-R9P in cultured human cells. (**A–D**) Various concentrations of the compounds were added to the cell culture media of U87-MG glioblastoma cancer cells at time point zero. 10 to 50 cells were evaluated in each experiment. Average values ± s.e.m. are shown. (**A**) 2-OG analogue dm2-OG; (**B**) Citrate; (**C**) Glutamine; (**D**) Glutamine following pre-incubation with 100 µM of the glutaminolysis inhibitor epigallocatechin gallate (EGCG). (**E**) Embryonic kidney cells (HEK293T) treated with dm2-OG. (**F**) Retinal pigment epithelium cells (RPE1) treated with dm2-OG.

As a proof of concept, we tested the TC3-R9P sensor (with a dynamic range between 10 µM – 10 mM) in the human glioblastoma cell line U87-MG. Deregulation of 2-OG metabolism in glioblastoma and other gliomas previously has been shown to be responsible for the aberrant epigenetic gene regulation that characterizes many of these tumors^16^. While healthy brain cells show 2-OG concentrations in the range of 1–3 mM, brain cancer cells contain drastically reduced concentrations ranging from 100–300 µM^16, 54^. Accordingly, we transiently transfected these cells with plasmids expressing TC3-R9P under the control of the cytomegalovirus (CMV) promoter. We measured the FRET change after adding either dm2-OG, citrate or glutamine in different concentrations to the cells. The cell-permeable compound dm2-OG is converted into 2-OG inside the cells^55^. As shown in Fig. 6, the FRET dropped within minutes after addition of the compounds, in a concentration-dependent manner. For example, with 20 mM dm2-OG, a 10% drop in FRET was observed **(**Fig. 6A; Supplementary Video 1 and 2 **)**. Theoretically, this drop could be explained by the direct effect of dm2-OG on the sensor; however, since TC3-R9P has an almost two orders of magnitude lower affinity for dm2-OG than 2-OG **(**Fig. 2D **)**, and dm2-OG furthermore is rapidly demethylated by esterases in the cell, this FRET change likely represents the accumulation of 2-OG in the cells.

Even more pronounced than dm2-OG, addition of citrate rapidly induced a 15% FRET drop, which is close to the maximal observable FRET change **(**Fig. 6B **)**. As the sensor is 3600 times less sensitive for citrate than for 2-OG, the strong drop in FRET can only be generated by the fast conversion of citrate to 2-OG by the TCA cycle. To estimate the absolute changes in 2-OG, we harvested cells 5 minutes after addition of citrate and determined 2-OG amounts by ultra-performance liquid chromatography. Relative to control cells which contained 56.12 ± 4.08 pmol/10^5^ cells (mean ± s.e.m.), addition of 5 mM and 10 mM citrate increased the 2-OG contents to 59.84 ± 1.95 (7%) and 67.77 ± 1.28 pmol/10^5^ cells (21%), respectively. In contrast to dm2-OG addition, the response towards citrate is more dynamic: addition of 10 mM citrate results in a rapid but transient FRET response. This indicates that citrate is actively taken up and rapidly metabolized to 2-OG leading to a peak in intracellular 2-OG levels. Subsequently, the 2-OG concentrations inside the cells return back to equilibrium, as revealed by the sensor. By contrast, in the case of dm2-OG, the FRET signal remains at a decreased steady state level over the measuring time. This indicates that the diffusion of dm2-OG into the cells and subsequent demethylation to 2-OG leads to increased 2-OG levels, which remain constant, since passive dm2-OG diffusion maintains the novel steady state level of 2-OG.

The growth of glioblastoma and many other cancer cell lines is dependent on the uptake of glutamine, which is converted to 2-OG by glutaminolysis. This process involves the enzyme glutamate dehydrogenase 1 (GLUD1), a potential target for anti-cancer therapy, whose activity can be suppressed by the small molecule inhibitor epigallocatechin gallate (EGCG)^56^. To test whether the TC3-R9P sensor could detect the intracellular increase of 2-OG level due to increased glutamine uptake and conversion to 2-OG by glutaminolysis, we added 5 mM or 10 mM glutamine to U87 MG cells that were cultured in low-glutamine conditions (0.5 mM instead of 2 mM). Immediately following glutamine supplementation we observed a concentration-dependent drop of the FRET ratios suggesting elevated uptake of glutamine by the cells and its conversion to 2-OG **(**Fig. 6C **)**. After an initial transient over-accumulation, 2-OG levels returned to a new steady state within a few minutes. When glutaminolysis was blocked by pre-incubation with 100 µM EGCG for three hours, no change in FRET was observed **(**Fig. 6D) indicating that the additional intracellular 2-OG that accumulated in the inhibitor-free condition **(**Fig. 6C) indeed was derived from glutamine. These data show that our P_II_-protein-derived FRET sensors potentially could be used to detect phenotype-relevant changes of 2-OG concentrations in cancer cells, making them useful tools to monitor readout during metabolic drug screening. To test whether the 2-OG sensor also could be used in other types of cells, we measured FRET changes in cell lines that have been derived from embryonic (human embryonic kidney; HEK293T) and differentiated (retinal pigment epithelium; RPE1) tissues. In both cell lines FRET ratios decreased following the addition of dm2-OG **(**Fig. 6E and F **)** suggesting that the TC3-R9P sensor can be used to analyze 2-OG changes in various types of cells.

In summary, with these various applications we could demonstrate the functionality of our FRET based 2-OG sensor for different applications. The TC3 sensor performed very well for *in situ* GOGAT activity determination. For determining cellular 2-OG concentrations, the final goal was to create a sensor that sensitively reports fluctuating 2-OG concentration in living cells. For this purpose, the R9P variant of the TC3 sensor seems to be a very good starting point. This sensor variant allowed real-time live-cell imaging of 2-OG in cancer, embryonic and differentiated cells, and potentially could also be used as a readout assay in high-throughput drug screening.

For even more sensitive *in vivo* experiments, the maximal signal change of 25% upon 2-OG addition should be further improved by protein engineering of the FRET sensor, to achieve a more robust signal. As seen in the sensor development, small alterations can have a drastic, unpredictable influence on the FRET output. Accordingly, we suggest a directed evolutionary approach to further improve the FRET sensor. It has been shown before, that a random mutagenesis approach with a well-designed selection mechanism can strongly enhance the capabilities of a FRET sensor^57, 58^. In view of the emerging importance of 2-OG as effector molecule for central cellular processes, monitoring *in vivo* fluctuations of 2-OG will lead to a deeper understanding on the role of the metabolism on cell functions.

## Material and Method

### Cultivation of bacterial and human cell lines

Cyanobacterial strains were cultivated photoautotrophically at a constant illumination of 50 µmol photons m^−2^ s^−1^ in BG-11 medium^59^ supplemented with 5 mM NaHCO_3_ at 28 °C in flasks shaking at 120 rpm. As nitrogen source, either 17.65 mM NaNO_3_ or 5 mM NH_4_Cl, with 5 mM TES/NaOH; pH 8.2 (Roth), was added.

*Escherichia coli* K-12 substrain MG1655 cells were cultivated in M9 minimal medium containing: 48 mM Na_2_HPO_4_, 22 mM KH_2_PO_4_, 8.5 mM NaCl, 19 mM NH_4_Cl, 2 mM MgSO_4_, 0.1 mM CaCl_2_, 0.4% (w/v) glucose, 0.0001% (w/v) thiamin. Cultures of 100–200 mL volume were grown in 1 L wide-neck flasks at 180 rpm, using silicone sponge closures to ensure aerobic growth. Optical density was measured at 600 nm.

Human glioma cell line U87-MG was cultured at 37 °C, 90% air/10% CO_2_ in Dulbecco’s Modified Eagle’s medium (DMEM Sigma D5921) without phenol-red, 1000 mg/L glucose, supplemented with 10% FCS (Biochrom #S0115), 2 mM glutamine and 1% penicillin/streptomycin mix. HEK293T cells were cultured in RPMI 1640 Medium, 4500 mg/L glucose, supplemented with 10% FCS, 2 mM glutamine and 1% penicillin/streptomycin mix. hTERT-RPE1 cells were cultured in DMEM/F12 Medium, 4500 mg/L glucose, supplemented with 10% FCS and additional 1% glutamine. All cell lines were transfected with 0.8 µg/mL plasmid DNA using TransIt-LT1 reagent (Mirus). Dimethyl-2-oxoglutarate (dm-2OG, Sigma), citric acid (AppliChem) and glutamine (Gibco 25030) were diluted in DMEM buffered with HEPES solution. For GDH inhibition, cells were pre-incubated 3 h with 100 µM (−)-Epigallocatechin gallate (Sigma E4143).

### Cloning and Protein purification

Plasmids were constructed by PCR amplification of P_II_ and FP genes with primers containing overlapping regions. The PCR products were fused with an XbaI and HindIII double digested pASK-IBA3 vector (IBA GmbH, Göttingen, Germany) via isothermal, single-reaction DNA assembly following the protocol by Gibson *et al*.^60^. The sensor genes were amplified by standard PCR and cloned into the mammalian expression vector pcDNA3.1 using the BamHI and HindIII restriction sites. The complete sequences of the sensor genes are provided in the Supplementary Information.

P_II_ protein variants were overexpressed in *E. coli* RB9060 and purified as described earlier using Strep-tag affinity chromatography^28^. To remove contaminating proteins and degradation products the sensor proteins were further purified by size exclusion chromatography, using an Äkta purifier and a HiLoad 26/600 Superdex 200 prep grade column (GE Healthcare GmbH, Solingen, Germany). The running buffer contained 30 mM Tris (pH 7.5) and 175 mM NaCl. The proteins were stored in a buffer containing 50 mM Tris-HCl pH 7.8, 100 mM KCl, 5 mM MgCl_2_, 0.5 mM EDTA, 1 mM DTT and 50% glycerol (v/v) at −20 °C.

### *In situ* fdGOGAT Assay

*In situ* Fd-GOGAT activity was determined according to Marqués *et al*.^44^ and Kameya *et al*.^45^, with some modifications. For assaying the *in situ* Fd-GOGAT activity in *S. elongatus*, 4*10^8^ cells were collected and washed twice with PBS buffer containing 2.7 mM KCl, 1.5 mM KH_2_PO_4_, 137 mM NaCl, 8.1 mM Na_2_HPO_4_, pH 7.5 at 4 °C. The cell pellet was resuspended in 200 µL reaction buffer containing 20 mM Na_3_PO_4_ pH 7.2, 10 mM glutamine, 1 mM 2-OG and 5 mM methyl viologen. Cells were permeabilized by addition of 4.4 µL toluene and constant vortexing at 1400 rpm for 5 min at 32 °C. To insulate the reaction from atmospheric oxygen, the reaction mixture was covered by 500 µL paraffin oil. The reaction was started by addition of 5 mM sodium dithionite, gently mixing and incubating at 32 °C. Reaction aliquots (30 µL) were removed in regular intervals and the reaction was stopped by intensive shaking until the blue color disappeared, indicating oxidation of the electron donor methyl viologen. Aliquots were then centrifuged at 15,000 × g for 10 min at 4 °C and the 2-OG concentration in the supernatant was determined as described below. One unit of activity was defined as µmol 2-OG consumption per min.

### Cell Extraction for FRET Measurements

*E. coli* cultures (20 ml aliquots) were harvested at an optical density (OD_600_) of 0.5 by pipetting directly out of the shaking culture and mixing with 30 mL of the corresponding culture medium and 30 g ice for rapid quenching of the metabolism. Cells were pelleted by centrifugation at 15,000 × g for 8 min at 0 °C. The cell pellet was resuspended on ice with 94 µL 20% (v/v) ethanol. The cell suspension was transferred into a 2 mL sample tube and 406 µL MilliQ water was added to a final volume of 500 µL with a final ethanol concentration of 3.75% (v/v), as well as 6.25 µL toluene for membrane permeabilization, as described by Gomez Casati *et al*.^46^. The mixture was incubated for 8 min at 25 °C under shaking at 1,400 rpm and subsequently quickly cooled on ice and centrifuged at 14,000 × g for 5 min at 0 °C. The clear supernatant was transferred into a fresh tube and centrifuged as before. The supernatant was either directly used for FRET measurements or stored at −20 °C.

### Ultra Performance Liquid Chromatography (UPLC) analysis

For determination of intracellular 2-OG content in human glioblastoma cell line, 1 × 10^5^ cells were extracted with 200 μl cold 1 M perchloric acid. Insoluble material was removed by centrifugation for 10 min at 25.000 × g. For derivatization with DMB (1,2-diamino-4,5-methylendioxybenzene), 30 μl extract were mixed with 30 μl DMB derivatization reagent (5 mM DMB, 20 mM sodium hydrosulfite, 1 M 2-mercaptoethanol, 1.2 M HCl) and incubated at 100 °C for 45 min. After 10 min centrifugation, the reaction was diluted with 240 μl 10% acetonitrile. The derivatized ketoacids were separated by reversed phase chromatography on an Acquity HSS T3 column (100 mm × 2.1 mm, 1.7 μm, Waters) connected to an Acquity H-class UPLC system. Prior separation, the column was heated to 40 °C and equilibrated with 5 column volumes of solvent A (0.1% formic acid in 10% acetonitrile) at a flow rate of 0.55 ml/min. Baseline separation of DMB derivates was achieved by increasing the concentration of acetonitrile (B) in buffer A as follows: 2 min 2% B, 4.5 min 15% B, 10.5 min 38% B, 10.6 min 90% B, hold for 2 min, and return to 2% B in 3.5 min. The separated derivates were detected by fluorescence (Acquity FLR detector, Waters; excitation: 367 nm; emission: 446 nm) and quantified using ultrapure standards (Sigma). Data acquisition and processing was performed with the Empower3 software suite (Waters). 2-OG data were globally normalized relative to the mean of all analyzed derivatized metabolites.

### *In vitro* FRET Measurements

FRET measurements were conducted in a buffer containing 50 mM imidazole (pH 7.5), 50 mM KCl, 20 mM MgCl2, 15% glycerol (v/v), 0.5 mM DTT and 5 mM ATP. The sensor protein was carefully added to a final concentration of 100 nM (monomer). For blank measurements the master mix was prepared by adding water instead of the sensor protein. Without mixing, the solution was incubated for 15 min on ice to allow the protein to adapt to the buffer. After that, the solution was mixed by inverting the tube several times and 70 µL (extract measurement) or 90 µL (GOGAT measurement) aliquots were dispensed onto a black non-binding 96-well plate (Greiner Bio-One, Frickenhausen, Germany). 30 µL of the cell extracts or 10 µL of the GOGAT assay supernatant and a 2-OG standard solution were added to the plate. The plate was sealed using an adhesive foil to prevent evaporation. Subsequently the plate was placed in a Tecan Spark 10 M microplate reader (Tecan, Männedorf, Switzerland) at 37 °C. The plate was shaken for 80 s and incubated for 20 min. After the incubation, the plate was again shaken for 20 s, the foil was removed and the FRET signal was measured. Each well was measured in three channels (each 20 nm bandwidth): Venus, excitation: 485 nm, emission: 530 nm; Cerulean: excitation: 435 nm, emission: 480 nm; FRET: excitation: 435 nm, emission: 530 nm. Blank values were subtracted from the measurements in each channel. Following the N_FRET_ formula developed by Xia and Liu^61^ corrected FRET values were calculated. The correction factors *a* and *b* were determined in separate measurements containing only Venus or Cerulean. Each plate contained a standard curve of known 2-OG concentrations and the samples. Using GaphPad Prism 6 (GraphPad Software, San Diego, USA) the unknown concentrations were interpolated from the standard curve. For the calculation of intracellular 2-OG concentrations the intracellular volume of glucose grown *E. coli* MG1655 was assumed to be 3.3 µL per mL of OD_600_ = 1 culture, as determined by Volkmer and Heinemann^62^.

### Life-Cell Imaging

Fluorescence measurements were performed in a Leica TCS-SP5 laser scanning confocal microscope equipped with resonant scanners and hybrid photon detectors (HyD) and using a 40x objective (HCX PL APO, NA 1.25, oil-immersion). Cells were excited with a UV diode (405 nm) and emission was detected at 450–490 nm (CFP) and 520–590 nm (FRET). Pictures were acquired every 60 sec. After a baseline of 5 min, media containing metabolites or not were added and changes in fluorescence were recorded for 15 minutes. During imaging, cells were maintained at 37 °C and 5% CO_2_. Image processing was performed with ImageJ (http://fiji.sc/). After background subtraction and registration, single cells were segmented by threshold and mean intensity was measured for each channel at every time point. The ratio was calculated by dividing the mean intensity of the acceptor (FRET) by the intensity of the donor (CFP). Ratios calculated from every cell were normalized to baseline measurements.

## Electronic supplementary material

Supplementary Info

Video 1 control treatment

Video 2 dm2-OG treatment
