# Supplementary material for: PII Protein-Derived FRET Sensors for Quantification and Live-Cell Imaging of 2-Oxoglutarate
Source: Sci Rep. 2017 May 3;7:1437. doi: 10.1038/s41598-017-01440-w (PMC5431102; doi:10.1038/s41598-017-01440-w)
Supplement: Supplementary file 1 — Supplementary Info [file 41598_2017_1440_MOESM1_ESM.pdf]

Supplementary Information

P_{II} Protein-Derived FRET Sensors for Quantification and Live-Cell Imaging of 2-Oxoglutarate

Jan Lüddecke[1§], Liliana Francois[2§], Philipp Spät[1¶], Björn Watzer[1¶], Tomasz Chilczuk[1], Gernot Poschet[3], Rüdiger Hell[3], Bernhard Radlwimmer[2], Karl Forchhammer[1]*

[1]Interfaculty Institute for Microbiology and Infection Medicine, Division Organismic Interactions, University of Tübingen, Tübingen, Germany

[2]Division of Molecular Genetics, German Cancer Research Center (DKFZ), Heidelberg, Germany

[3]Centre for Organismal Studies Heidelberg, Rupprecht-Karls-Universität Heidelberg, Heidelberg, Germany

Supplementary Methods

Gas Chromatography - Mass Spectrometry Determination of 2-OG Concentrations

Gas Chromatography - Mass Spectrometry (GC-MS) measurements were performed according to Fiehn et al. In brief, 80 μL of the extracts and 2-OG standard solutions were lyophilized and subsequently resuspended in 25 μL methoxamine hydrochloride solution (20 mg/mL in pyridine) and incubated for 90 min and 30 °C shaking at 1400 rpm to protect the carbonyl moieties. The samples were then silylated by addition of 25 μL N methyl-N-trimethylsilyltrifluoroacetamide (MSTFA) and incubation for 30 min at 37 °C and further 2 h at RT. For the GC/MS based quantification of derivatized 2-OG, 1 μL of the solution was applied to an Agilent 6890A gas chromatograph online coupled to an Agilent 5973N mass spectrometer. The sample was injected splitless at an injection temperature of 280 °C onto a HP-5MS column with the dimensions of 30 m × 250 µm. Helium was used as carrier gas with a flow rate of 1 mL/min at 0.6 bar. Chromatography was carried out with the following temperature gradient: 5 min solvent delay time at constant 70 °C, increase to 170 °C with 5 °C/min and from 170 °C to 290 °C with

30 °C/min. 290 °C was kept for additional 10 min followed by cooling-down to 70 °C. This temperature was kept for additional 6 min. Analytes were online transferred to the MS at 280 °C and ionized in EI mode. Temperatures of the MS source and the MS quadrupole were set to 230 °C and 150 °C, respectively. Data were acquired in Selected Ion Monitoring mode (SIM) for fragment ions m/z 147 and 198. For 2-OG quantification, integrated peak areas corresponding to m/z 198 (at 24.01 min) were used. Based on the 2-OG standard solution calibration series a linear function was determined which was used to calculate the 2-OG concentrations of the respective samples.

Fiehn, O., Kopka, J., Trethewey, R. N. & Willmitzer, L. Identification of uncommon plant metabolites based on calculation of elemental compositions using gas chromatography and quadrupole mass spectrometry. *Anal Chem* **72**, 3573-3580 (2000).

FRET Sensor sequences

Complete nucleotide sequence of the PII-TC3 sensor:

ATGAAGAAGATTGAGGCGATTATTCGTCCGTTCAAACTGGACGAAGTCAAGATTGCGCTCGTCAATGCT
GGCATTGTCGGGATGACGGTTTCAGAAGTGCGCGGTTTTGGTCGCCAAAAAGGCCAAACGGAGGCCATC
AGCGACAACGTCTATATCACCGCCGACAAGCAGAAGAACGGCATCAAGGCCAACTTCAAGATCCGCCA
CAACATCGAGGACGGCAGCGTGCAGCTCGCCGACCACTACCAGCAGAACACCCCCATCGGCGACGGCC
CCGTGCTGCTGCCCGACAACCACTACCTGAGCACCCAGTCCAAGCTGAGCAAAGACCCCAACGAGAAG
CGCGATCACATGGTCCTGCTGGAGTTCGTGACCGCCGCCGGGATCACTCTCGGCATGGACGAGCTGTAC
AAG*GGTTCTGGTGGTACCGGT*GTGAGCAAGGGCGAGGAGCTGTTCACCGGGGTGGTGCCCATCCTGGT
CGAGCTGGACGGCGACGTAAACGGCCACAAGTTCAGCGTGTCCGGCGAGGGCGAGGGCGATGCCACC
TACGGCAAGCTGACCCTGAAGTTCATCTGCACCACCGGCAAGCTGCCCGTGCCCTGGCCCACCCTCGTG
ACCACCCTGACCTGGGGCGTGCAGTGCTTCGCCCGCTACCCCGACCACATGAAGCAGCACGACTTCTTC
AAGTCCGCCATGCCCGAAGGCTACGTCCAGGAGCGCACCATCTTCTTCAAGGACGACGGCAACTACAA
GACCCGCGCCGAGGTGAAGTTCGAGGGCGACACCCTGGTGAACCGCATCGAGCTGAAGGGCATCGACT
TCAAGGAGGACGGCAACATCCTGGGGCACAAGCTGGAGTACAACCGCTATCGCGGCTCGGAATACACG
GTTGAGTTTTTGCAAAAGCTGAAGCTCGAGATCGTGGTGGAAGATGCCCAAGTCGACACCGTCATCGAC
AAAATCGTTGCAGCAGCCCGCACTGGCGAAATTGGTGACGGCAAGATCTTCGTCTCGCCCGTCGACCAA
ACCATCCGGATTCGCACCGGCGAGAAAAACGCCGACGCAATC*AGCGCTTGGAGCCACCCGCAGTTCGA*
***AAAATCT*GCGGTGAGCAAGGGCGAGGAGCTGTTCACCGGGGTGGTGCCCATCCTGGTCGAGCTGGACG**
GCGACGTAAACGGCCACAAGTTCAGCGTGTCCGGCGAGGGCGAGGGCGATGCCACCTACGGCAAGCTG
ACCCTGAAGCTGATCTGCACCACCGGCAAGCTGCCCGTGCCCTGGCCCACCCTCGTGACCACCCTGGGC
TACGGCCTGCAGTGCTTCGCCCGCTACCCCGACCACATGAAGCAGCACGACTTCTTCAAGTCCGCCATG

CCCGAAGGCTACGTCCAGGAGCGCACCATCTTCTTCAAGGACGACGGCAACTACAAGACCCGCGCCGA
GGTGAAGTTCGAGGGCGACACCCTGGTGAACCGCATCGAGCTGAAGGGCATCGACTTCAAGGAGGACG
GCAACATCCTGGGGCACAAGCTGGAGTACAACTACAACAGCCACAACGTCTATATCACCGCCGACAAGC
AGAAGAACGGCATCAAGGCCAACTTCAAGATCCGCCACAACATCGAGGACGGCGGCGTGCAGCTCGCC
GACCACTACCAGCAGAACACCCCCATCGGCGACGGCCCCGTGCTGCTGCCCGACAACCACTACCTGAG
CTACCAGTCCGCCCTGAGCAAAGACCCCAACGAGAAGCGCGATCACATGGTCCTGCTGGAGTTCGTGAC
CGCCGCCGGGATCACTCTCGGCATGGACGAGCTGTACAAGTAA

The colors indicate sequence segments encodig parts of the sensor:

- **red underlined font:** **PII**
- **blue font:** **mCerulian**
- ***grey italic font*** **: mCerulian linker region**
- ***green italic font*** **: Strep Tag**
- **orange font:** **Venus protein**

Supplementary Figures

Supplementary Figure S1

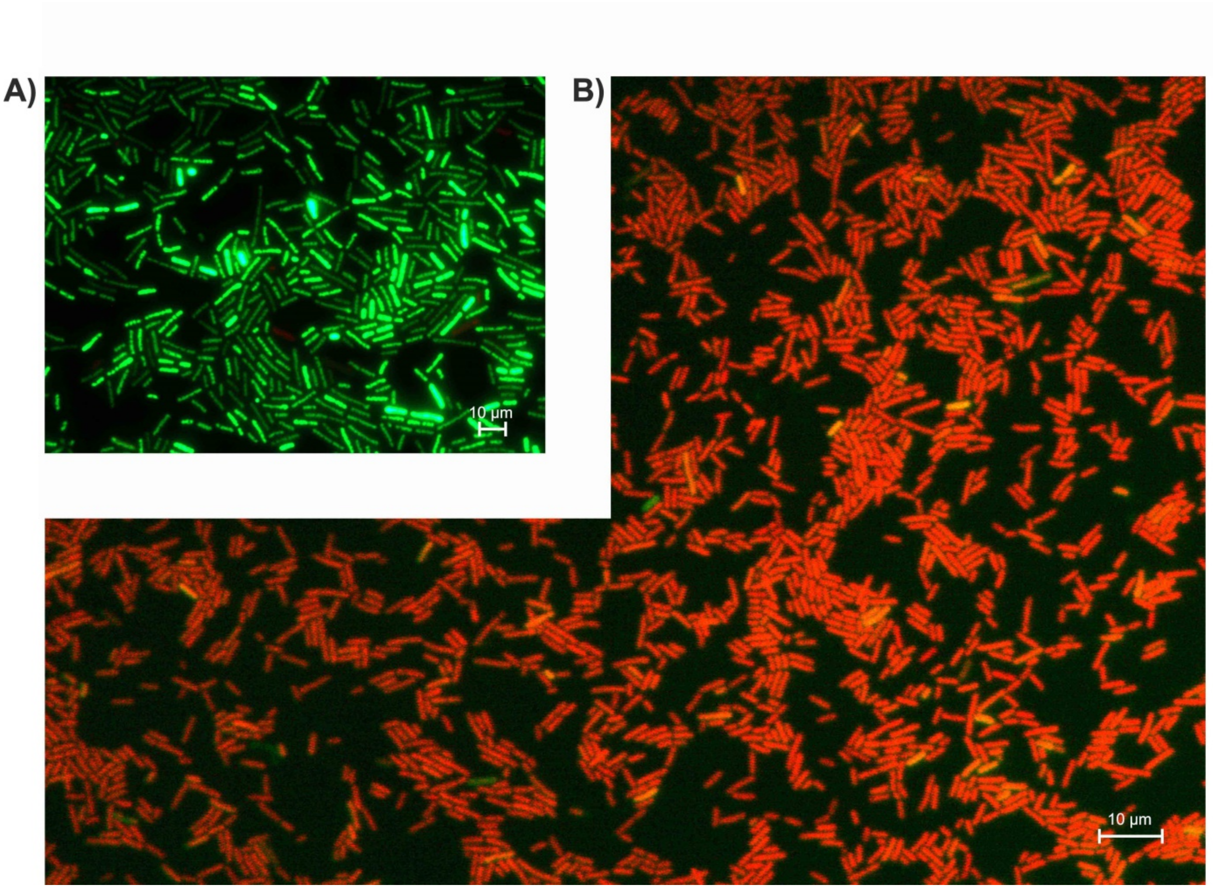

Figure S1. Permeabilization efficiency test via live/dead staining.
The cells were treated according to the manual of the live/dead BacLight™ bacterial Viability kit (Thermo Fisher). The kit contains two nucleic acid stains: SYTO® 9 (Green) and propdium iodide (red). SYTO® 9 can penetrate both alive and dead bacteria. In contrast, propdium iodide penetrates only bacterial cells with damaged membranes and quenches SYTO® 9 fluorescence. Hence, the green fluorescence indicates intactness of the cell membrane while the red auto-fluorescence indicates damaged membranes. For fluorescence imaging a Leica DM2500 microscope with a Leica DFC420C color camera was used. **(A)** *S. elongatus* cells before toluene permeabilization. **(B)** *S. elongatus* cells after toluene permeabilization as described in the methods section.

Supplementary Figure S2

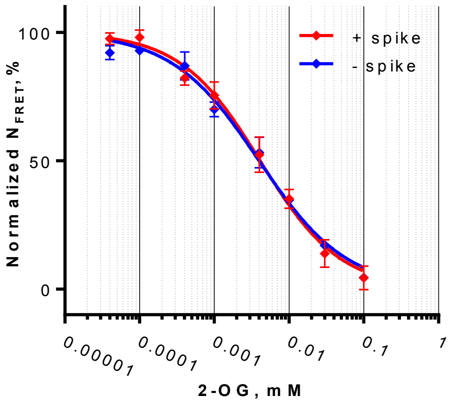

Figure S2. Influence of the GOGAT assay components on the FRET-sensor.
The influence of the GOGAT assay components on the performance of the FRET-sensor was tested by measuring two 2-OG standard curves. While one FRET master mix was supplemented with the GOGAT assay mix without 2-OG (red) the control experiment was prepared with water instead of GOGAT assay mix (blue).

Supplementary Video 1 and 2

Time-lapse imaging of 2-OG concentrations in U87MG cells using the TC3-R9P sensor. Ratiometric changes of intracellular 2-OG after addition of 0 mM (video 1) and 20 mM dm-2-OG (video 2) are shown. The pseudocolor scale bar indicates the ratios of FRET and CFP emission intensities.
